# Osteosarcoma in Pediatric and Adult Populations: Are Adults Just Big Kids?

**DOI:** 10.3390/cancers15205044

**Published:** 2023-10-19

**Authors:** Caleb Kim, Lara E. Davis, Catherine M. Albert, Brian Samuels, Jesse L. Roberts, Michael J. Wagner

**Affiliations:** 1 Division of Hematology and Oncology, University of Washington, Spokane, WA 99202, USA; ckim428@uw.edu; 2 Division of Hematology/Medical Oncology, Knight Cancer Institute, Oregon Health & Science University, Portland, OR 97239, USA; 3 Division of Pediatric Hematology, Oncology, Bone Marrow Transplant and Cellular Therapy, Seattle Children’s Hospital, Seattle, WA 98105, USA; 4 Beacon Cancer Care, Coeur d’Alene, ID 83814, USA; blsamuels.md@gmail.com; 5 Department of Orthopaedics and Sports Medicine, University of Washington, Seattle, WA 98109, USA; 6 Division of Hematology and Oncology, University of Washington, Seattle, WA 98109, USA; 7 Clinical Research Division, Fred Hutchinson Cancer Center, Seattle, WA 98109, USA

**Keywords:** osteosarcoma, bone tumors, AYA, adolescent/young adult oncology, sarcoma

## Abstract

**Simple Summary:**

Osteosarcoma is an aggressive cancer of the bone that can present in children or adults. It has historically been thought that adults have worse clinical outcomes than children. However, current treatment strategies for adults are largely extrapolated from pediatric studies since the majority of clinical trials for osteosarcoma treatments are based on younger patient populations. We summarize the current understanding of the molecular etiology of osteosarcoma and how it may differ between age groups, hypothesizing why adult patients have worse outcomes compared to children.

**Abstract:**

Malignant bone tumors are commonly classified as pediatric or adolescent malignancies, and clinical trials for these diseases have generally focused on these populations. Of primary bone cancers, osteosarcoma is among the most common. Osteosarcoma has a bimodal age distribution, with the first peak occurring in patients from 10 to 14 years old, and the second peak occurring in patients older than 65, with about 25% of cases occurring in adults between 20 and 59 years old. Notably, adult osteosarcoma patients have worse outcomes than their pediatric counterparts. It remains unclear whether age itself is a poor prognostic factor, or if inherent differences in tumor biology exist between age groups. Despite these unknowns, current treatment strategies for adults are largely extrapolated from pediatric studies since the majority of clinical trials for osteosarcoma treatments are based on younger patient populations. In light of the different prognoses observed in pediatric and adult osteosarcoma, we summarize the current understanding of the molecular etiology of osteosarcoma and how it may differ between age groups, hypothesizing why adult patients have worse outcomes compared to children.

## 1. Introduction

Osteosarcoma can occur in any bone of the body, but classically develops in the metaphysis of the long bones around the knee in the distal femur and the proximal tibia, and the proximal humerus [1]. It has a bimodal distribution of incidence among age groups, with a first peak of incidence in children and adolescents and a second peak in those over 65 years old [1]. Although osteosarcoma is generally considered a pediatric sarcoma, the majority of patients are older than 25 years old [2]. These patients are underrepresented in clinical trials, which have historically focused on the pediatric and adolescent/young adult (AYA) populations, while excluding adults over 40 from eligibility [3,4]. Most studies that do include older adult patients have shown worse outcomes when compared to children on the same treatment regimen (Table 1) [5,6,7,8]. Yet still, the treatments developed from these studies have been extrapolated to guide the management of adults [9,10]. 

Whether age alone represents an independent prognostic factor for osteosarcoma remains uncertain. Large groups such as the Children’s Oncology Group (COG), Cooperative Osteosarcoma Study Group (COSS), and the Scandinavian Sarcoma Group (SSG) have reported long term survival rates of predominantly pediatric osteosarcoma patients (inclusion age < 40 years old) of over 70% [3,11,12]. Comparatively, adults from 30 to 49 years old have survival rates as low as 50% [5,13]. A multivariate analysis of 172 patients at the Norwegian Radium Hospital showed that patients older than 40 had significantly worse outcomes, and that an age over 40 had utility as an independent prognostic risk factor [14]. A larger study of 665 high-grade osteosarcoma patients at the Korea Cancer Center Hospital also found lower survival rates for patients over 40 years old when compared to adolescent patients, citing unusual tumor locations, difficulty with surgery, and poor responses to chemotherapy as reasons for the difference [15]. Among the largest studies is an analysis of 11,961 cases of osteosarcoma through the National Cancer Database of the American College of Surgeons, which reported a clear inverse relationship between age and 5-year survival rates, with a 60% 5-year survival rate for patients younger than 30 years old, 50% for patients between 30 and 49 years old, and just 30% for those above 50 years old [13]. Additionally, adult patients have higher rates of metastatic relapse. A study of 1054 patients from North American cooperative group trials demonstrated that adults over 18 with osteosarcoma have a higher rate of disease recurrence, which is associated with a significant decrease in overall survival [4]. Lower survival rates and higher rates of relapse in adult osteosarcoma patients are primary indicators that the disease affects certain age groups differently.

In contrast to these studies showing that advanced age correlates with worse outcomes for osteosarcoma, at least some studies have suggested that other age-independent factors might account for the differences. Specifically, a meta-analysis including 4838 patients from five international cooperative groups identified age as a significant prognostic factor with children having improved overall survival compared to adolescents and adults, but this difference was not significant in multivariate analysis [16]. Similarly, an analysis of a cohort of 438 patients at the M.D. Anderson Cancer Center found that variables such as tumor necrosis, tumor size, and tumor location are more significant factors in determining overall survival and disease-free survival, and that age was not an independent prognostic factor [17]. An analysis of 1702 patients with high-grade osteosarcoma from the Cooperative German-Austrian-Swiss Osteosarcoma Study Group found that older age was not associated with outcomes in a multivariate model, despite univariate analysis suggesting that it was a negative prognostic indicator [7], and age similarly did not impact the survival outcomes in a large series from a single center in Italy [18,19]. One of the only prospective trials on osteosarcoma in patients above the age of 40, EURO-BOSS, suggests that adults over 40 and younger patients may even share similar survival rates with aggressive chemotherapy and surgery [20]. However, the 208 adult patients in this trial had higher rates of significant chemotherapy-related toxicities, including peripheral neuropathy and nephrotoxicity, despite receiving lower doses of methotrexate compared to younger patients [20]. To explore why adults have different outcomes than children, we herein examine the etiology of osteosarcoma, discussing the known distinctions between pediatric and adult patients. Although there is no clear consensus for what age cutoff defines an adult, for the purposes of our discussion, we will generally consider adults as those above the age of 40.

**Table 1:** Survival by age group in selected prospective clinical studies.

Regimen	Age Range	Survival		Reference
MAP vs. MAP/IE	40 years or younger (does not report by age group)	-Mean time to first event 43.3–44.1 months -3 year EFS 53–55%		Marina et al., 2016 [3]
Multiple regimens on COG trials CCG-7943, POG-9754, INT-0133, and AOST0121	<10 10 to 17 ≥18	10-year EFS 55% 55 37	10-year OS 68% 60 41	Janeway et al., 2012 [4]
MAP based chemo	Child (0–11/12) * Adolescent (12–17) Adult (≥17)	HR (EFS primary endpoint) 1.00 1.25 1.32		Smeland et al., 2019 [5]
High-dose methotrexate, Adriamycin, and BCD	<20 ≥21	DSS, % at 5, 10, 15 years 34, 31, 31 40, 27, 19		Bernthal et al., 2012 [6]
Multiple regimens included	<40 ≥40	10-year OS 60.2% 41.6%		Bielack et al., 2002 [7]
Multiple regimens included	<15 ≥15	10-year OS 76% 68%		Fuchs et al., 1998 [8]
MAP vs. MAP/IE	<40	5-year OS 74%		Smeland et al., 2003 [12]
Multiple regimens included	Child (0–11/12) * Adolescent (12–17) and Adult (≥17)	HR (OS from study entry) 1.0 1.23		Collins et al., 2013 [16]
Doxorubicin, cisplatin, ifosfamide, and methotrexate suggested	41–65	5-year OS 66%		Ferrari et al., 2018 [20]

* Age ranges differed by sex. DSS, Disease specific survival. MAP = Methotrexate, Adriamycin, Cisplatin; IE = ifosfamide and etoposide. BCD = bleomycin, cytoxan, actinomycin D.

## 2. Differences in Chemotherapy Tolerance

One simplistic potential explanation for the suboptimal survival in older patients is that they are less likely to tolerate the intensive regimens of chemotherapy that have become standard in the pediatric population [21,22]. Current chemotherapy regimens most often employ cisplatin, methotrexate, and doxorubicin, with ifosfamide sometimes included as a fourth agent. Many of these chemotherapeutics have side effects that manifest more severely in older patients. For example, cisplatin is dose-limited by its nephrotoxicity. For older patients, there is a significant increase in risk of renal cell death and acute kidney injury [23,24]. Methotrexate similarly presents with an increased risk of kidney injury. Overall renal function declines with older age; therefore, methotrexate dosages are reduced or it is omitted entirely to limit toxic drug accumulation and kidney damage [20,25,26]. Other notable age-related toxicities include cardiotoxicity from doxorubicin treatment, with older patients experiencing significantly increased risks of congestive heart failure and cardiomyopathy [27,28]. In addition to more severe organ toxicities, chemotherapy-induced myelosuppression, including neutropenia, is more common in older patients and can lead to life-threatening infectious complications. To minimize these risks in adults, there are dose reductions and delays in standard treatment, which potentially compromise optimal treatment outcomes when compared to young patients, who can more readily adhere to the intensive chemotherapy protocols [29].

The significant side effects can compound the perception among older patients that chemotherapy is associated with adverse effects on multiple aspects of life, adding to an overall hesitancy to undergo treatment. A small survey of oncology nurses found that from the nurses’ perspective, older age may be associated with higher rates of chemotherapy discontinuation both due to side effect tolerance and social issues such as arranging transportation and other logistical aspects of receiving chemotherapy [30]. These perceptions by adult patients and the possibility that they may prefer approaches focusing on quality of life may diminish treatment results and be a strong confounding factor when considering age as a diagnostic variable.

## 3. Differences in Underlying Tumor Presentation

### 3.1. Tumor Location

Tumor location is an important prognostic factor for osteosarcoma. Axial tumors have significantly worse outcomes compared to appendicular tumors [16]. One hypothesis for this difference by tumor location is that it is due to the increased technical difficulty of surgical resection, leading to higher rates of incomplete resection and local recurrence [21,31]. Tumors that arise in the trunk are also more difficult to detect, leading to delayed diagnosis and prolonging the time until treatment, which are associated with larger tumor volumes and an increased likelihood of metastases. These factors may help to explain why patients with axial primary tumors have worse outcomes compared to tumors in other locations [7]. In the Euramos trial, axial location had no impact on survival for patients achieving complete surgical remission, suggesting no difference in the tumor biology between axial- and extremity-located tumors [5]. A recent analysis of patients with osteosarcoma in the US National Cancer Database separated patients into axial osteosarcoma, appendicular osteosarcoma, and lesions in other primary sites. Patients with tumors in the axial bones have worse 1-year, 5-year, and 10-year survival rates when compared to patients with tumors in the appendicular skeleton [32]. Patients with osteosarcoma in the axial skeleton also have a higher average age [32]. Analysis of the SEER database likewise reported a relationship between tumor location and age, with 25–59-year-old patients having a higher frequency of osteosarcoma in the axial skeleton (24.7%) compared to 0–24-year-old patients (8.1%) [2]. One explanation for why these cases are more common in younger patients is that appendicular bones experience the most rapid bone growth during puberty [33]. Rapidly proliferating osteogenic cells are especially prone to mitotic errors and chromosomal rearrangement, leading to overall chromosomal instability and the development of osteosarcoma [34]. In adults, axial tumors are more common due to association with previous irradiation and rare diseases like Paget’s disease of bone, which correlate with older age [2,21]. Since these patterns can be distinguished by age, they may be indicative of an inherent difference in tumor biology by age and suggest that there are different drivers of mutation and tumorigenesis, which will be discussed in subsequent sections.

### 3.2. Primary versus Secondary Osteosarcoma

Compared to pediatric osteosarcomas, adult osteosarcomas are more likely to be secondary osteosarcomas associated with previous radiotherapy or, in some cases, with Paget’s disease of bone [22]. Paget’s disease is a disorder of bone metabolism that is characterized by hyperactive cells of the bone, with larger osteoclasts that have more nuclei and osteoblasts that form bone matrices more rapidly and in a disorganized manner, producing its characteristic “mosaic” pattern [35,36]. The development of osteosarcoma in patients with Paget’s is rare (1–2%), but this still represents a several thousand-fold increase in risk compared to the general population [37]. Paget’s associated osteosarcomas occur in older age and are found along the axial skeleton, contributing to its dismal prognosis, with a 5-year survival rate of only 10% [38]. While the etiology of Paget’s disease and the way in which it causes osteosarcoma remains unknown [39], this cohort of adult patients contributes to the overall inferior outcome in adults. 

Previous radiation exposure is another cause of secondary osteosarcoma in adults, often occurring many years after the radiation was delivered [40,41]. Osteosarcoma is the most common radiation-induced bone tumor, representing 50–60% of cases [42]. Radiation-induced bone sarcomas are associated with increased morbidity and mortality compared with primary bone tumors [41,43]. Radiation-induced osteosarcoma is associated with a 50% lower 5-year overall survival when compared to primary cases of osteosarcoma [44]. Hypothesized reasons for worse prognoses include late diagnosis, advanced tumor grade at diagnosis, tumor location, and, for cases where complete surgical resection is not feasible, the inability to prescribe full-dose postoperative radiotherapy in a previously irradiated field [45]. Due to fears of the serious side effects caused by re-irradiation and limited efficacy data for re-irradiation, few patients with radiation-associated sarcomas receive radiation therapy [45,46]. There is also evidence of an increased incidence of TP53 somatic mutations, leading to a loss of heterozygosity in radiation-induced sarcomas [47]. 

Secondary osteosarcomas disproportionately affect adults and are associated with poorer outcomes than de novo osteosarcomas; thus, secondary osteosarcomas likely contribute to poor overall outcome estimations for adult osteosarcoma. 

## 4. Differences in Underlying Tumor Biology (Summarized in Figure 1)

### 4.1. Chromosomal Instability

Genomic alterations in osteosarcoma are complex, with heterogenous patterns of genomic rearrangement and mutation [48]. Some genes, including FLCN, CCND3, and HSP90AB1, are more frequently altered in pediatric versus adult osteosarcoma [49]. However, no single underlying mutation clearly leads to osteosarcoma. Rather, the disease is characterized by genomic and chromosomal instability manifested as the gain or loss of entire chromosomes or sections of chromosomes, associated with copy number alterations and ineffective DNA damage response mechanisms [50]. Causes of instability include the mutation or deregulation of genes important for mitotic checkpoints, with TP53 and RB mutations as important examples. Other sources of instability include the loss of telomere regulation, catastrophic chromosomal events, and miRNA regulation. We examine each of these causes of instability and point out distinctions by age group.

**Figure 1:**
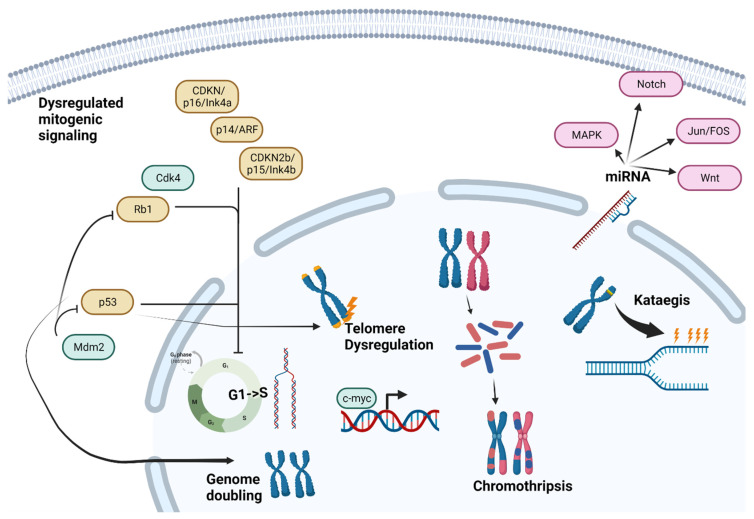
Osteosarcoma cell alterations potentially contributing to age-related differences in outcomes. Genes/proteins in green are amplified or pathologically activated, genes/proteins in yellow are tumor suppressors, and pathogenic alteration is loss of function. Abbreviations: Cdk4, Cyclin Dependent Kinase 4; Rb1, RB Transcriptional Corepressor 1; p53, Tumor Protein P53; Mdm2, MDM2 Proto-Oncogene; CDKN/p1/Inka, Cyclin Dependent Kinase Inhibitor 2A; p14/ARF, ARF tumor suppressor; CDKN2b/p15/Inkb, Cyclin Dependent Kinase Inhibitor 2B; MAPK, Mitogen-Activated Protein Kinase pathway; Jun/Fos, Jun/Fos Proto-oncogene pathway; Wnt, Wnt/Beta-catenin pathway.

### 4.2. Mitogenic Signaling and Cell Cycle Checkpoints

Defects in the genes involved in mitotic checkpoints are established contributors to chromosomal instability (summarized in Supplemental Table S1). The tumor suppressor protein p53, encoded by TP53, is important in cell cycle arrest in response to DNA damage, inducing cellular quiescence, senescence, or apoptosis [51]. The loss of TP53 precedes an alternative lengthening of telomeres events (ALT, further discussed below) and whole genome doubling events, contributing to overall genomic instability [52]. In an analysis of the targeted next generation sequencing of 765 osteosarcoma patients, patients younger than 30 were found to have a 3.8% frequency of carrying a germline mutation associated with Li-Fraumeni syndrome (LFS) or a likely LFS-associated mutation, compared with no LFS-associated mutations in patients older than 30 (n = 51) in this series [53]. The somatic whole genome sequencing of 34 osteosarcoma samples from patients younger than 18 revealed that > 90% of the tumors had mutations or structural variations in the TP53 gene [54]. Although common in osteosarcoma across age groups, TP53 abnormalities appear more common in children younger than 18, as whole genome sequencing studies inclusive of patients over 18 report a 40–75% frequency of TP53 pathway mutations [55,56]. The loss of the heterozygosity of the gene locus 17p13.1, where the TP53 gene is located, is detected in 29–42% of cases of sporadic osteosarcoma [57]. In addition to the direct inactivation of TP53, the functional inactivation of p53 at the post-translational level can lead to the deregulation of the TP53 pathway. The MDM2 protein promotes the degradation of p53 and downregulates its transcription [50]. It also affects the RB pathway through the direct binding and promotion of Rb protein degradation [58]. The amplification of MDM2 on chromosome 12q15 has been detected in 16% of osteosarcomas [59]. The aforementioned p14/ARF gene on chromosome 9p21 has also been implicated in the inhibition of MDM2 function and the poor prognosis in osteosarcoma patients [60]. Another site of amplification that is of interest is chromosome 17p11.2-p12. It leads to the increased expression of COPS3, which has an important role in promoting the degradation of p53 and is found in 31% of osteosarcoma cases [61].

As mentioned above, the retinoblastoma (RB) pathway is another important source of mitotic checkpoint alterations in osteosarcoma [62]. The RB1 gene, located at 13q14.2, encodes the tumor suppressor protein Rb, which prevents the progression of the cell cycle following the detection of DNA damage [63]. In patients with germline RB1 mutations, the incidence of osteosarcoma increases up to 500 times that of the general population [64,65]. Somatic RB1 alterations are present in about half of the sporadic cases of osteosarcoma, most often as deletions [56,66]. In addition to direct alterations of RB1, there are RB1-independent mechanisms that lead to RB pathway deregulation. The amplification of CDK4 on chromosome 12q, a kinase that phosphorylates the RB product to promote cell cycle progression from G1 to S, is detected in about 10% of tumors [67]. Genomic loss frequently occurs in regulators of CDK4, such as CDKN2A/p16/INK4A, p14/ARF, and CDKN2B/p15/INK4B [68]. These mutations are all located on chromosome 9p21, which undergoes deletion in 5–21% of cases of osteosarcoma [68]. The cases of osteosarcoma secondary to germline RB pathway mutations show a younger age of onset, at about 11 years old, suggesting that this pathway of tumorigenesis primarily affects pediatric patients [69,70]. 

Alterations in c-MYC on chromosome 8q24.21 is an important emerging prognostic factor that is implicated in chromosomal instability in osteosarcoma [71]. c-MYC overexpression is associated with higher rates of metastases, and shorter metastasis-free survival time in osteosarcoma [72]. MYC inactivation has been studied as a therapeutic target, as its inactivation induces tumor cells to undergo terminal differentiation into mature bone cells [73]. In a study of 56 osteosarcoma tissue samples not identified by age, the positive immunohistochemical expression of c-MYC was observed in 85.7% of samples [74]. Interestingly, one study found amplifications of the c-MYC gene in just 7% of adult cases of osteosarcoma, while another study reported a 40% frequency in childhood osteosarcoma compared with 5% in adults [75,76]. 

Although some gene alterations involved in cell cycle progression seem more prevalent in younger populations, not all studies have found significant molecular differences by age in osteosarcoma. Specifically, an effort to compare the NGS results of 67 patients aged between 8 and 80 found no significant differences in the molecular alterations or gene mutations between pediatric (18 or less) or adult cases [77]. A larger collaborative effort is needed to confirm if true molecular differences in osteosarcoma by age exist. 

### 4.3. Telomere Dysregulation

The loss of telomere regulation is another source of chromosomal instability in cancer, including osteosarcoma. Germline mutations affecting telomere function disproportionately predispose carriers to sarcomas over carcinomas [78]. The alternate mechanism of telomere maintenance (ALT), which is defined by heterogenous and elongated telomeres without telomerase activity, is one mechanism by which sarcoma cells avoid senescence [79,80]. ALT is observed in ~5–15% of all cancers, but it is more prevalent in osteosarcoma, seen in up to 48% of cases [79,81]. Osteosarcomas with germline shelterin complex mutations, which are critical for normal telomere regulation [82], can develop secondary somatic mutations in related genes such as SMARCAL1 and STAG3 [78]. In contrast, Mirabello et al. showed that single nucleotide polymorphisms (SNPs) in TERF1, a component of the shelterin nuclear protein complex that functions as a telomerase inhibitor, protect against osteosarcoma development. Telomere length inversely correlates with age, but age did not seem to correlate with overall osteosarcoma risk in this study; females with shorter telomeres may have an increased osteosarcoma risk [83]. Increased telomerase activity in primary osteosarcoma tumors correlates with worse progression-free and overall survival [84], which is consistent with the finding that a genetic predisposition to longer telomere length at birth may increase the overall risk of developing osteosarcoma [85]. However, a genetic predisposition to longer telomere length does not clearly correlate with age at the diagnosis of osteosarcoma [86]. Therefore, in spite of the associations with age and telomere length and the separate association between telomere regulation and the risk of osteosarcoma development, aberrant telomere regulation does not clearly explain any differences in the outcome for patients with osteosarcoma by age. 

### 4.4. Chromosomal Phenomena

Chromothripsis is a genetic phenomenon leading to genomic instability, where chromosomes are shattered into hundreds to thousands of pieces by ionizing radiation, radio-mimetic chemicals, or DNA replication errors, then rejoined by DNA repair mechanisms, often inaccurately. In contrast to a gradual accumulation of localized mutations, chromothripsis is a singular cellular catastrophe that leads to clustered chromosomal rearrangements prone to tumorigenesis [87,88]. It is seen in 2–3% of all cancers, but strikingly, it is seen in up to 77% of cases of osteosarcoma [88]. In a study of 34 osteosarcoma tumor samples, chromothripsis events were associated with recurrent mutations in the TP53, RB1, MYC, and PTEN pathways, alongside mutations in ATRX, LSAMP-AS3, CCNE1, COPS3, PMP22, MAPK7, NCOR1, and UBB [54,62]. In a whole genome sequencing and molecular profiling study of 48 pediatric and adult osteosarcoma resected tumor specimens, tumors from younger patients displayed greater levels of focal clustered rearrangements, suggesting a higher incidence of chromothripsis events [52]. In contrast, Behjati et al. identified chromothripsis amplification and other genomic alterations with similar frequencies in the osteosarcomas of different age groups [89]. 

Another important phenomenon associated with chromosomal instability is kataegis, or regional hypermutation. Kataegis is defined as a pattern of single nucleotide variant clusters with five shared mutational signatures [90]. It is observed in 50–85% of cases of osteosarcoma [54,56]. In other types of cancer, such as breast cancer, kataegis is associated with a later age of diagnosis [91]. Similar to telomere dysregulation, these chromosomal phenomena are shared characteristics between age groups in patients with osteosarcoma and are not known to correlate with osteosarcoma outcomes.

### 4.5. miRNA Expression

miRNAs can operate as oncogenes and tumor suppressor genes, and have been observed to regulate specific tissue lineages during osteosarcoma differentiation [92,93]. The post-transcriptional miRNA regulation of intracellular signaling pathways associated with osteosarcoma have been identified, including NOTCH, Ras/p21, MAPK, Wnt, and the Jun/FOS pathways [94]. Osteosarcoma patients younger than 20 years old had higher levels of miR-497, a miRNA involved in hypoxia response by suppressing hypoxia-inducible factor 1-α (HIF-1α), and older patients (>20 years old) had lower levels of hsa-miR-203, a miRNA with tumor-suppressive properties that is correlated with worse survival, but is not known to impact HIF-1 α [95]. HIF-1α is known to be pro-inflammatory and promote cancer processes, and this differential miRNA expression may lead to a more tumorigenic microenvironment by preferentially promoting angiogenesis, proliferation, and metastasis in older patients [96].

## 5. Immune Microenvironment

Although therapies targeting the immune microenvironment have revolutionized cancer care in recent years, immunotherapy has shown limited activity in osteosarcoma [97]. In a study of 48 osteosarcoma tumor samples from both children and adults, there was evidence of differing levels of tumor immunosuppression between age groups [52]. When classified into three clusters based on hierarchical clustering from low to high, the majority of patients aged older than 50 years (9/14) were in the group with the highest immune infiltration, including CD8 lymphocytes. However, the samples with higher immune infiltration also had higher levels of immunosuppressive markers, including PD-L1, CTLA4, and IFNG signaling, as well as myeloid-derived suppressor cells [52]. Adults older than 45 had higher PDL1 expression and lower B-cell abundance than younger patients [49]. In soft tissue sarcomas, the presence of B-cells and tertiary lymphoid structures is a strong favorable prognostic indicator [98]; their relative paucity in adult osteosarcoma may suggest less intrinsic immunogenicity in those tumors. Adult osteosarcomas have a higher tumor mutation burden (TMB) than children, but this difference is unlikely to be clinically significant as the TMB is generally low for all cases [77]. Overall, the balance (or imbalance) skewing towards an immunosuppressive phenotype in older osteosarcoma patients may suggest that older patients have an overall reduced ability for T-cell activation and an immune suppressive microenvironment, which may in part contribute to poor outcomes.

## 6. Other Heritable Cancer Predisposition Syndromes

In addition to germline RB1 and TP53 mutations (discussed above in “Mitotic Checkpoints”), several other rare inherited syndromes are associated with osteosarcoma development and can differentially affect older or younger age groups.

Rothmund–Thomson syndrome (RTS) is a rare autosomal recessive genetic disorder associated with pathogenic variants of the RECQL4 gene on chromosome 8q24.4, which codes for a DNA helicase in the RecQ family [99,100]. Another RECQL4-related syndrome is RAPADILINO syndrome, which is characterized by several major clinical findings and predominantly occurs in Finland. A study in Finland found that 13.3% of RAPADILINO syndrome patients developed osteosarcoma [101]. Each of these RECQL4 diseases are associated with younger ages of onset for osteosarcoma [101,102,103,104].

Bloom and Werner syndromes also involve the RecQ DNA helicase family. Bloom syndrome is a germline BLM (RECQL3) inactivation located at chromosome 15q26.1, and is most common in people of Eastern European Jewish ancestry [105]. Werner syndrome is a germline WRN (RECQL2) inactivation located at chromosome 8p12, found most commonly in Japan, and usually diagnosed in the fourth decade of life [106]. Both are autosome recessive syndromes that predispose patients to osteosarcoma. Like the previously mentioned predisposition syndromes, Bloom syndrome is associated with a younger age of onset of cancers, including osteosarcoma [107]. Werner syndrome has the opposite correlation, being associated with an older age of onset of osteosarcoma [108].

Two other extremely rare syndromes that are associated with an increased incidence of osteosarcoma are ATR-X syndrome, which is also characterized by developmental delay and alpha-thalassemia, and Diamond-Blackfan anemia, an autosomal dominant congenital red cell aplasia that presents in infancy and early childhood [109,110,111]. Both are associated with younger ages of osteosarcoma development [109,110].

## 7. Treatment Implications

With the uncertainty of age as a prognostic factor, we continue to treat adults with multi-agent chemotherapy regimens, usually excluding methotrexate for most patients older than 40 for tolerability concerns. The data reviewed here highlight the importance of consensus guidelines with multi-institutional and even multi-national collaboration. The osteosarcoma community has demonstrated that international collaboration for clinical trials is feasible with several large-scale clinical trials, and this success in clinical collaboration must be extended to translational science.

Despite widespread and enthusiastic interest, targeted therapy has yet to have a significant impact on the survival of osteosarcoma patients, whereas, at least in the pediatric population, intensive combination cytotoxic chemotherapy has been game-changing. While future research into new targets is important and essential, with the relatively new ability to conduct and coordinate larger scale clinical trials in rare tumors on an international scale, there might also be an opportunity to conduct clinical trials evaluating the use of newer, less toxic chemotherapy agents, such as aldoxorubicin or L-annamycin, specifically in adult osteosarcoma populations to test the concept that it is primarily a lack of toleration of intensive chemotherapy, which has limited survival for adult osteosarcoma patients.

Newer agents targeting some of the driver alterations may be especially relevant for patients > 40 years, where standard poly-agent chemotherapy is less tolerated and optimal dose intensity will rarely be obtained. MDM2 inhibitors are currently being studied in multiple cancer types and may present an opportunity to target TP53 wild-type osteosarcomas [112]. CDK4/6 inhibitors have also shown some promise in the preclinical setting for osteosarcoma with intact Rb and may be combined with standard chemotherapy [113]. A clinical trial combining the G2-M checkpoint Wee1 inhibitor ZN-c3, combined with gemcitabine, is ongoing and enrolling both pediatric and adult subjects (NCT04833582) [114]. Ultimately, a large collaborative basket trial without age limit bounds (lower or upper) is an approach that can allow for an assessment of rationally targeted therapy for each individual patient, regardless of age.

With more immune infiltrates and a slightly higher TMB, one might postulate that rational combinations incorporating immunotherapy may be more effective for adult osteosarcoma. Clinical trials with immunotherapy in osteosarcoma have limited success, although rare responses in adults are seen [97]. With chemotherapy tolerability concerns in older adults and a suggestion that adult osteosarcoma may be more immunogenic, prospective immunotherapy trials focusing on older adults may be informative.

## 8. Conclusions

**Figure 2:**
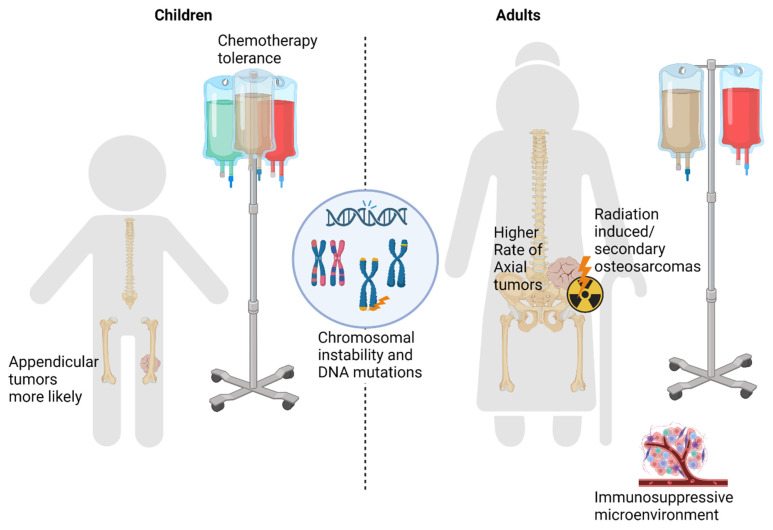
Differences and Similarities in Osteosarcoma by Age.

In summary, although older patients with osteosarcoma are known to have worse outcomes compared to children, age as an independent prognostic factor has not been firmly established. Several factors, including differences in tumor presentation, the ability to receive optimal chemotherapy, and differences in underlying tumor biology may play a role in the divergent outcomes between children and adults (Figure 2). Axial tumor location and tumors secondary to previous radiotherapy are known poor prognostic features that are common in adult osteosarcoma. A lower tolerance to chemotherapy prevents older patients from adhering to the protocol standards used in pediatric patients, producing suboptimal treatment response. These factors all correlate with older age and have been shown to have negative impacts on overall survival, helping to explain why adult osteosarcoma patients have worse outcomes. Other biologic features of osteosarcoma can be used to categorize pediatric and adult patients. The TP53, RB, and c-MYC mitotic checkpoints are more commonly altered in osteosarcoma in younger patients,. Chromothripsis is a catastrophic genomic event seen more commonly in tumors in young patients, while kataegis may be more characteristic of tumors in older patients. A more immunosuppressive microenvironment in adult tumors may also contribute to the observed differential outcomes by age group. Heritable predisposition syndromes mostly impact younger patients. While these factors individually do not explain the etiology of osteosarcoma, when taken together, they outline key distinguishing features between pediatric and adult osteosarcomas that may guide future translational medicine and prospective clinical investigation.

## Supplementary Materials

The following supporting information can be downloaded at: https://www.mdpi.com/article/10.3390/cancers15205044/s1, Table S1. Selected References for Mitogenic Signaling and Cell Cycle Checkpoint Alterations in Osteosarcoma.
