# Supplementary material for: Osteosarcoma in Pediatric and Adult Populations: Are Adults Just Big Kids?
Source: Cancers (Basel). 2023 Oct 19;15(20):5044. doi: 10.3390/cancers15205044 (PMC10604996; doi:10.3390/cancers15205044)
Supplement: Supplementary file 1 [file cancers-15-05044-s001.zip › cancers-2631796-supplementary.pdf]

**Supplemental Table S1**. Selected References for Mitogenic Signaling and Cell Cycle Checkpoint Alterations in Osteosarcoma

| Molecular Alteration | N | Key findings | Reference |
|---|---|---|---|
| *TP53* alterations | 765 | < 30 years: 3.8% with germline mutations<br>>30 years : 0% with germline mutations | Mirabello et al., 2015 |
| | 34 | ≤ 18 years: >90% with TP53 alterations<br>No adults included in this study. | Chen et al., 2014 |
| | 25 | 18 samples with somatic alterations (ages included 8-79), not reported by age | Lorenz et al., 2016 |
| | 59 | 12% had germline TP53 variants<br>75% had TP3 inactivation | Perry et al. 2014 |
| MDM2 / CDK4 amplification | 207 | 16% osteosarcoma samples in pan-cancer study with MDM2 amplification, no specification of age of patients | Momand et al., 1998 |
| | 34 | ≤18 years: 3% with structural variant in MDM2<br>No adults included in this study. | Chen et al., 2014 |
| | 66 | 14% with MDM2 or CDK4 amplification | Suehara et al., 2019 |
| *RB1* | | Germline *RB1* mutations confers 400-500x risk of osteosarcoma over general population | Wong et al., 1997 |
| | 59 (ages 6-28) | 3% with somatic RB1 mutations, 61% with RB1 deletion | Perry et al., 2014 |
| | 34 | ≤18 years: 29% with somatic RB1 alteration<br>No adults included in this study. | Chen et al, 2014 |
| | 72 samples (from 67 total patients)<br>n=33, age range 8–18<br>n=34, age range 19–80 | 19.4% of all samples with RB1 alterations, no difference between children or adults | Suehara et al, 2019 |
| | 30 | 43% with somatic RB1 alterations (8 patients 18 or older, oldest 42) | Toguchida et al, 1988 |
| *MYC* | 56 | 85.7% of all tumors with c-MYC expression by IHC | Wu et al., 2012 |
| | 25 | Amplification in 7% adults | Ladanyi et al., 1993 |
| | 258 | Amplification in 40% children and 5% adults | De Noon et al., 2021 |
